# Supplementary material for: Linking post-translational modifications and protein turnover by site-resolved protein turnover profiling
Source: Nat Commun. 2022 Jan 10;13:165. doi: 10.1038/s41467-021-27639-0 (PMC8748498; doi:10.1038/s41467-021-27639-0)
Supplement: Supplementary file 1 — Supplementary Information [file 41467_2021_27639_MOESM1_ESM.pdf]

Linking post-translational modifications and protein turnover by site-resolved protein turnover profiling

Jana Zecha[1,2,3], **Wassim Gabriel**[1], **Ria Spallek**[4,5], **Yun-Chien Chang**[1], **Julia Mergner**[1], **Mathias Wilhelm**[1], **Florian Bassermann**[2,4,5], **Bernhard Kuster***[1,2,6]

[1] *Chair of Proteomics and Bioanalytics, Technical University of Munich (TUM), Freising, Germany*

[2] *German Cancer Consortium (DKTK) and German Cancer Research Center (DKFZ), Heidelberg, Germany*

[3] *Current address: Dynamic Omics, Centre for Genomics Research, Discovery Sciences, R&D AstraZeneca, Gaithersburg, Maryland, United States*

[4] *Department of Medicine III, Klinikum rechts der Isar, TUM, Munich, Germany*

[5] *TranslaTUM, Center for Translational Cancer Research, TUM, Munich, Germany*

[6] *Bavarian Biomolecular Mass Spectrometry Center (BayBioMS), TUM, Freising, Germany*

* *Corresponding author (kuster@tum.de)*

Supplementary Information

- Supplementary Figures 1-9
- Supplementary Discussion
- Supplementary References

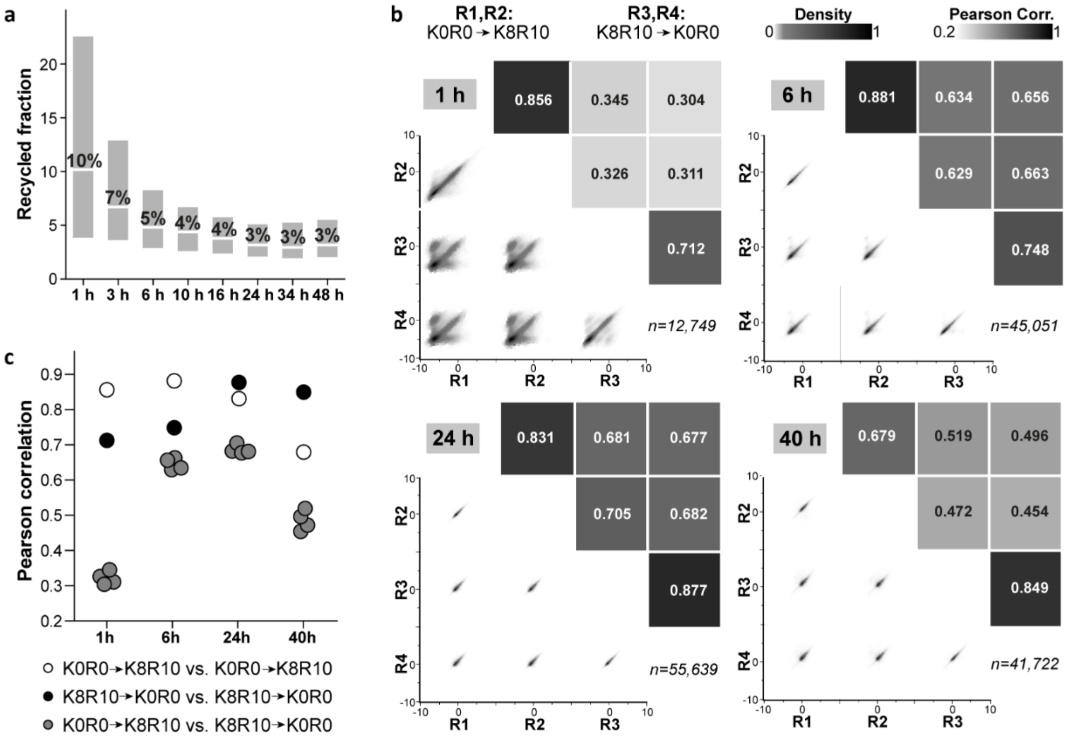

Supplementary Fig. 1 | Individual time-points using the dynamic SILAC strategy. a Rate of amino acid recycling for different pulse lengths. The recycling rate was calculated using peptides with one missed cleavage site that were quantified in a mixed SILAC labelling state and simultaneously in the labelling state representing peptides originating from newly synthesized proteins. Floating bars indicate the 25th to 75th percentile of recycling rate distributions. Medians are indicated. **b** Density multi-scatter plots for 4 cell culture replicates of 4 individual dSILAC time-points including label swap experiments. Log2 transformed ratios of newly synthesized to pre-existing peptides are plotted, and Pearson correlation coefficients and number of included modified peptidoforms are shown. **c** Pearson correlation coefficients between cell culture replicates for individual pulse lengths. The poorer correlation between label swap experiments in the 1 h and 40 h pulse experiment indicates erroneous quantification for very short and very long pulses. Source data are provided as a Source Data file.

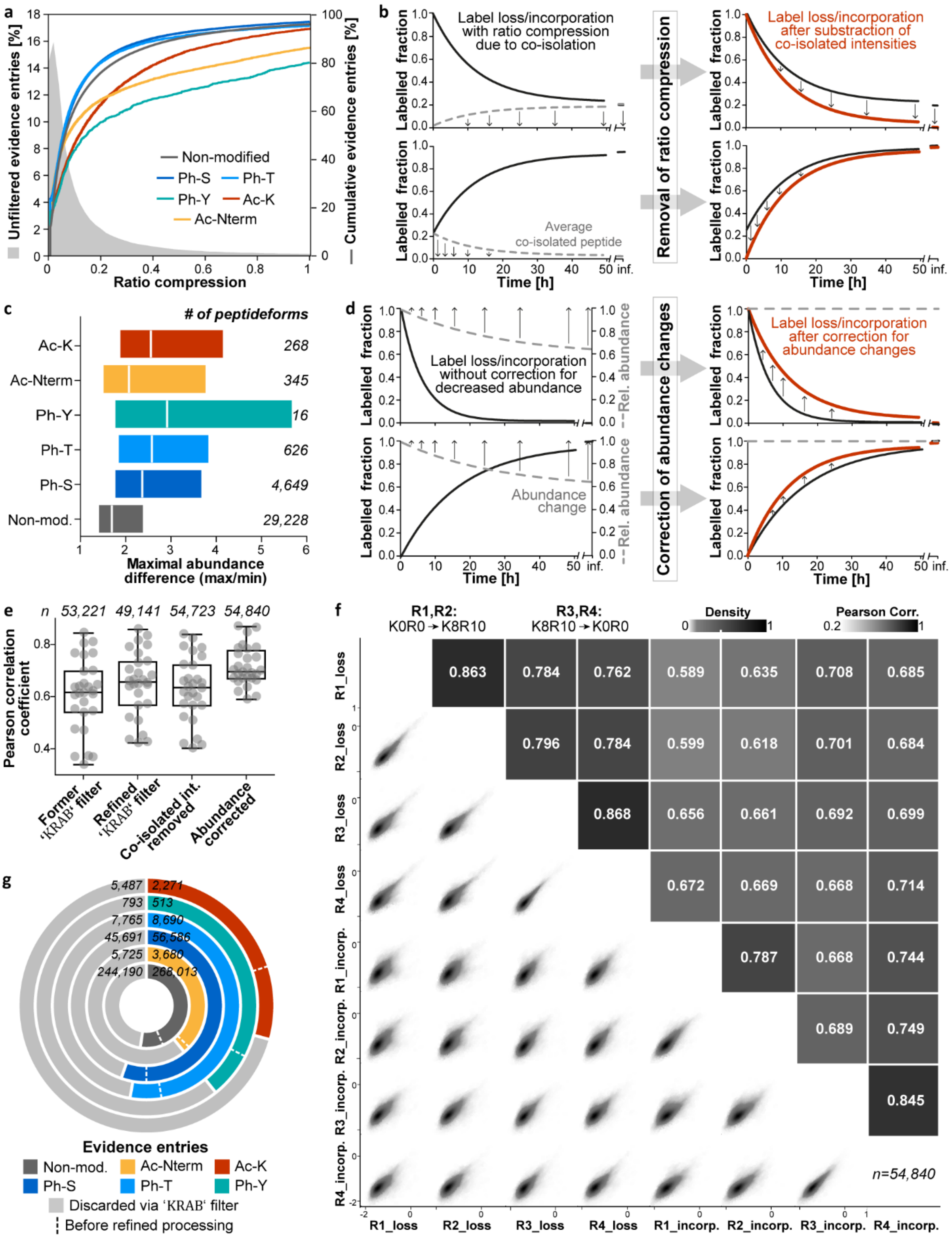
a
Unfiltered evidence entries [%]
Cumulative evidence entries [%]
Ratio compression
Non-modified
Ph-S
Ph-T
Ph-Y
Ac-K
Ac-Nterm
b
Label loss/incorporation with ratio compression due to co-isolation
Removal of ratio compression
Label loss/incorporation after substraction of co-isolated intensities
Average co-isolated peptide
Labelled fraction
Time [h]
c
# of peptideforms
Ac-K 268
Ac-Nterm 345
Ph-Y 16
Ph-T 626
Ph-S 4,649
Non-mod. 29,228
Maximal abundance difference (max/min)
d
Label loss/incorporation without correction for decreased abundance
Abundance change
Rel. abundance
Correction of abundance changes
Label loss/incorporation after correction for abundance changes
e
n 53,221 49,141 54,723 54,840
Pearson correlation coefficient
Former 'KRAB' filter
Refined 'KRAB' filter
Co-isolated int. removed
Abundance corrected
f
R1,R2: K0R0 → K8R10
R3,R4: K8R10 → K0R0
Density
Pearson Corr.
R1_loss
R2_loss
R3_loss
R4_loss
R1_incorp.
R2_incorp.
R3_incorp.
R4_incorp.
0.863 0.784 0.762 0.589 0.635 0.708 0.685
0.796 0.784 0.599 0.618 0.701 0.684
0.868 0.656 0.661 0.692 0.699
0.672 0.669 0.668 0.714
0.787 0.668 0.744
0.689 0.749
0.845
n=54,840
g
5,487 2,271
793 513
7,765 8,690
45,691 56,586
5,725 3,680
244,190 268,013
Evidence entries
Non-mod.
Ac-Nterm
Ac-K
Ph-S
Ph-T
Ph-Y
Discarded via 'KRAB' filter
Before refined processing

Supplementary Fig. 2 | Processing and quality of dSILAC-TMT data. a Distribution of ratio compression of all unfiltered evidence entries (area) and cumulative distributions of ratio compression for different modification states (lines). Ratio compression was defined as residual fractional intensity in TMT channels that should exhibit zero intensity by experimental design (TMT1 (0 h pulse) for entries showing label incorporation and TMT10 (∞ h pulse) for entries representing label loss). **b** Schematic illustrating the subtraction of co-isolated intensities to remove residual ratio compression computationally. **c** Distributions of maximal abundance changes during the pulse time-course for differentially modified peptidoforms. Floating bars display the 25th to 75th percentile. Medians (vertical lines) and number of peptides for which abundance changes could be calculated (numbers on the right) are indicated. **d** Schematic showing the principle underlying the correction for abundance changes. **e** Boxplots visualizing the changes of Pearson correlation coefficients for subsequent refinement steps of the processing of dSILAC-TMT data. Boxes represent the 25th to 75th percentile with the median in the middle, and whiskers show the range of data points. Log10 transformed turnover rates of peptidoforms were correlated. The number of sequences available for correlation analysis is displayed on top of each box plot. Following former/new 'KRAB' filter criteria were used: K: 0–5/0–5; $R \geq$ 0.8/0.7; A: 0.67–1.5/0.7–1.4; B: 0–0.3/-0.15–0.25. **f** Density multi-scatter plots for label loss and incorporation curves of 4 cell culture replicates including label swap experiments. Log10 transformed turnover rates are plotted, and Pearson correlation coefficients and number of included peptidoforms are indicated (corresponding to 'Abundance corrected' boxplot in panel e). **g** Doughnut plot illustrating the fraction of evidence entries passing the 'KRAB' curve filter before (dashed line) and after (solid line) removal of ratio compression and abundance correction for differentially modified peptides. The number of entries passing and filtered out after the refinement of data processing are indicated. Evidence entries were not considered for this analysis if they identified peptides in a mixed labelling state or showed zero intensities in more than eight time-points or in the '0 h' channel for label loss or the '∞ h' channel for label incorporation entries. Source data are provided as a Source Data file.

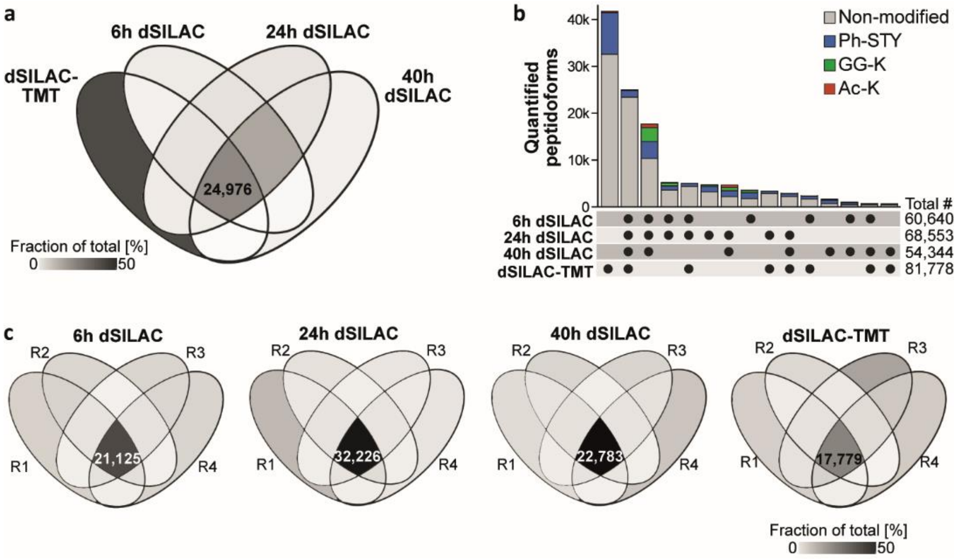

Supplementary Fig. 3 | Overlap of peptidoforms across datasets and replicates. For all comparison, oxidized versions of peptides were not counted separately. **a** Venn diagram showing the overlap of peptide sequences for the dSILAC-TMT and the three single dSILAC experiments. The colour gradient depicts the fraction of all peptidoforms in the respective overlap. **b** Bar chart illustrating the overlap and modification type of peptidoforms for the 4 different datasets. **c** Same as a, but displaying the overlap of peptide sequences across replicates for the 4 different datasets.

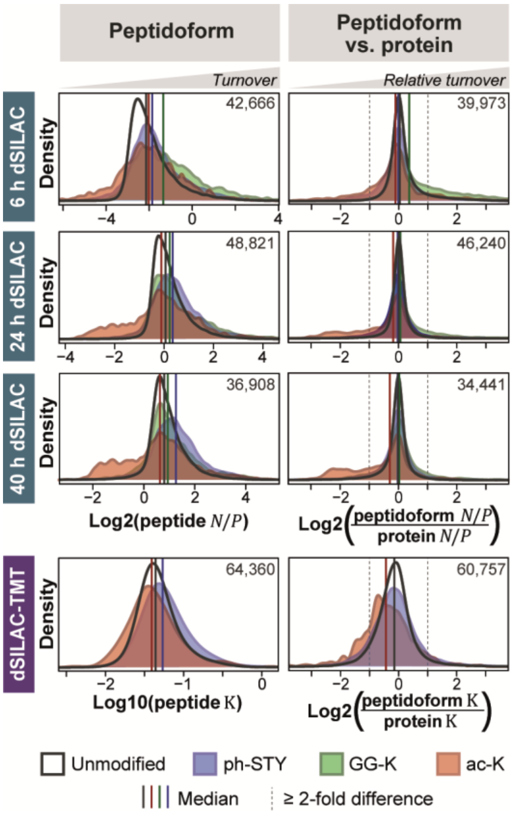

Supplementary Fig. 4 | Global associations of PTMs with protein turnover. Distributions of newly synthesized-to-pre-existing (N/P) ratios and turnover rates (K) for modified and unmodified peptidoforms (left column) and distributions of their N/P ratios and Ks relative to the N/P ratios and Ks of their corresponding proteins (right column). Ratios to proteins were calculated based on the median of log2 values across replicates. For the comparison to the protein turnover, only proteins with at least 3 peptides were included. Numbers of unmodified peptidoforms are given in the top right corner. For other numbers, see main fig. 2. Source data are provided as a Source Data file.

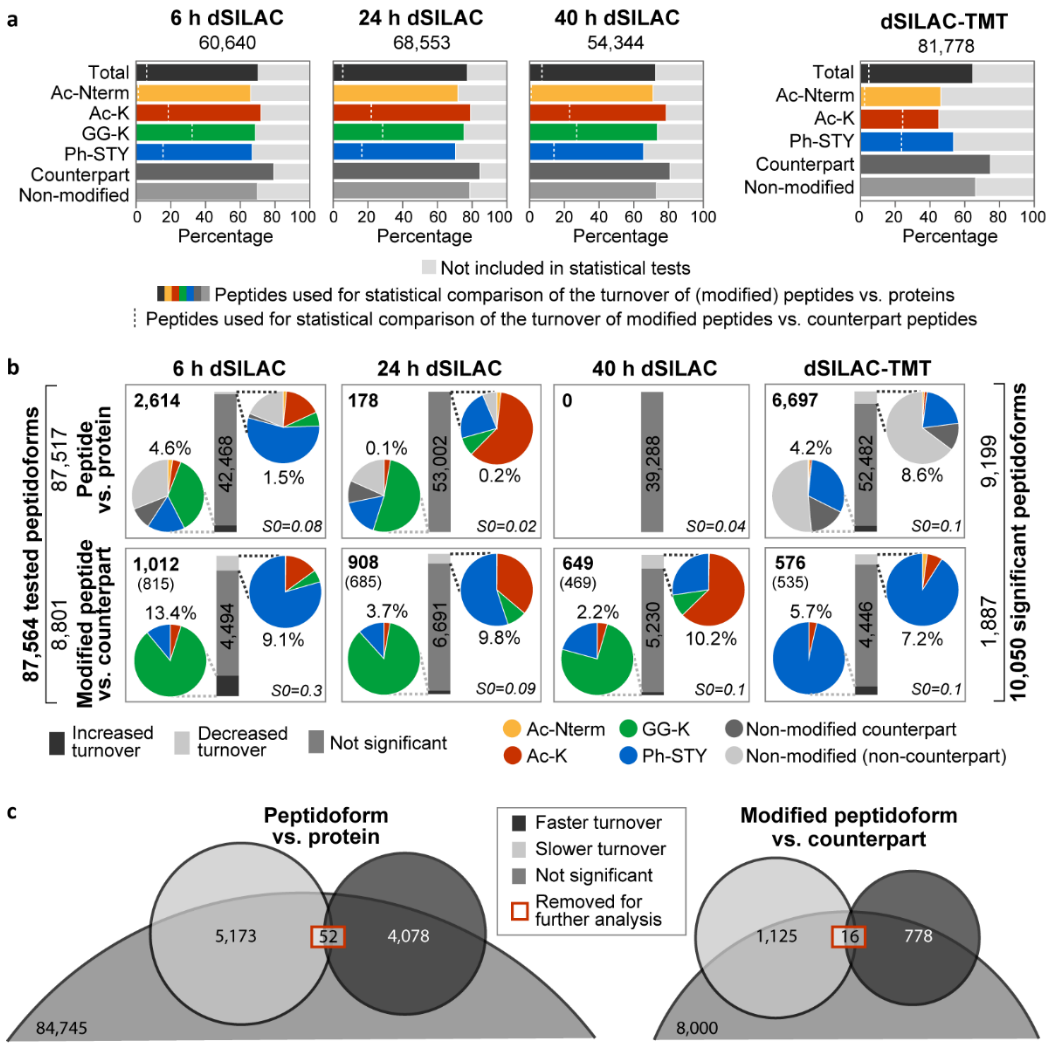

Supplementary Fig. 5 | Peptidoforms with significantly differential turnover. a Bar charts illustrating the fraction of quantified peptidoforms used to test significant turnover differences of (modified) peptides to their assigned proteins or their unmodified counterpart peptides. Peptides were excluded from statistical analyses if they featured less than 2 quantifications per statistical group or less than 3 quantified peptidoforms per assigned protein. The total number of peptides for each dataset is indicated on top. **b** Stacked bar charts and pie charts showing the fraction of peptidoforms with statistically significant turnover differences and their modification types. Significance of turnover differences between (modified) peptides and their proteins or counterpart peptides was tested for all datasets separately using log2 transformed data and two-sided Student's t-tests at 1 % permutation-based FDR. Respective S0 values are shown in italics. Numbers of pairwise comparisons (vertical numbers in bars) and fractions of significant peptide hits (percentages) are indicated for each of the 8 statistical analyses. Note that for the modified peptide-counterpart comparison, modified peptides can be included in the analysis more than once if several non-modified counterpart peptides were quantified. The total number of significant hits for each test is indicated in bold font, and the numbers of significant, distinct modified peptides for the modified peptide-counterpart tests are displayed in brackets. The total numbers of tested and significant peptidoforms are shown on the left and right side, respectively. **c** Weighted Venn diagrams displaying the number of peptidoforms that were identified with significantly faster or slower turnover compared to their protein (left site) or unmodified counterpart peptides (right side) in any of the 4 datasets. Peptidoforms that were found in more than one group (faster, slower, not significant) in t-tests were counted for every group producing the overlaps in the Venn diagram. Otherwise, numbers represent non-redundant peptidoforms. Peptidoforms that exhibited a significantly faster turnover and, in a different test, a significantly slower turnover (red boxes) were discarded from further enrichment analyses.

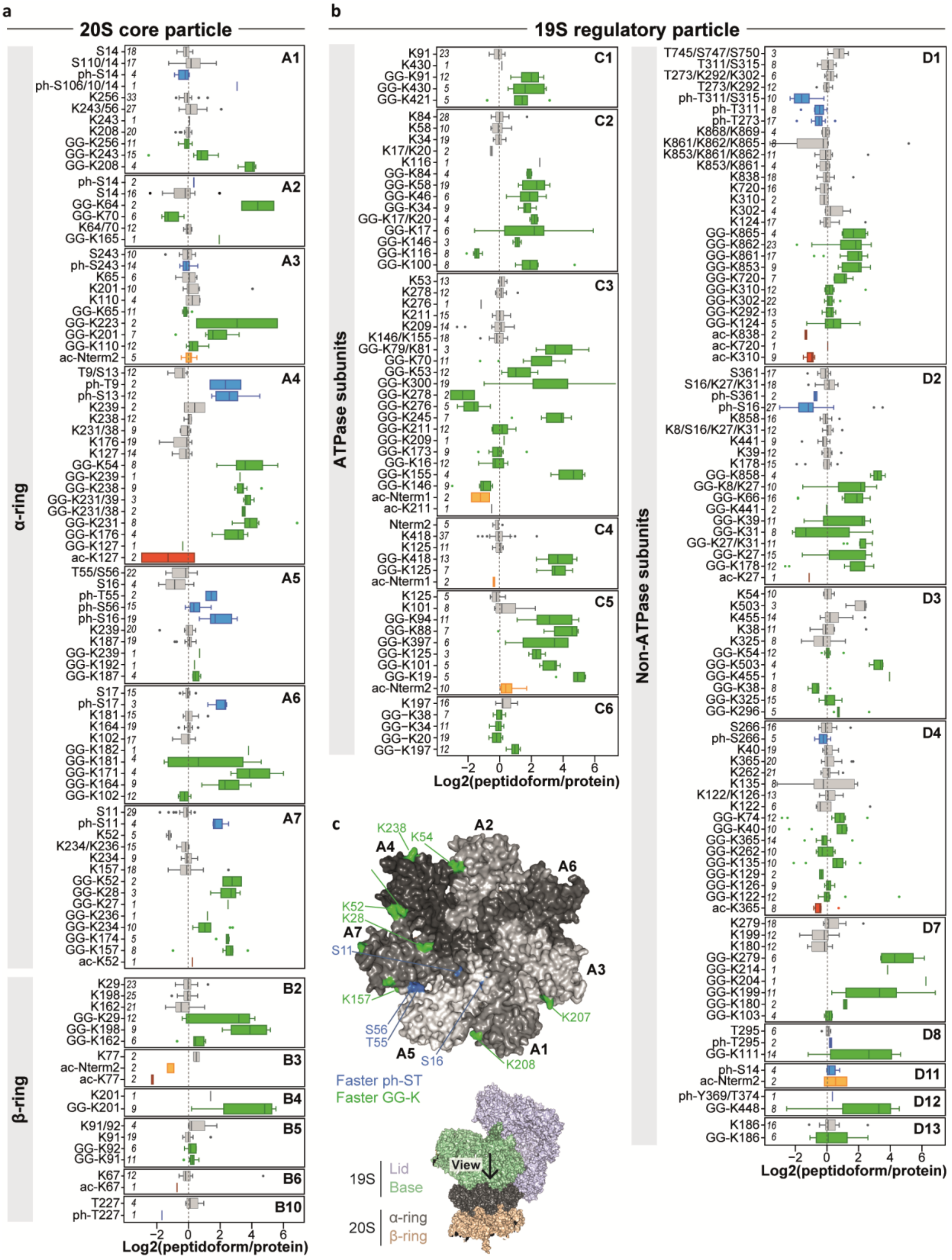

Supplementary Fig. 6 |. Measured PTM-resolved turnover for subunits of the 26S proteasome. a Tukey-boxplots of log2 turnover differences of all quantified PTMs and unmodified counterpart sites on 20S core subunits (box borders: 1st and 3rd quartile; lines in boxes: medians; whiskers: ranging to greatest value within 1.5× interquartile range; dots: outliers; dashed line: median of the total protein turnover). Each site includes all available data from peptidoforms mapping to that site in the three datasets. Number of data points is indicated in italics. Ratios of peptidoforms to corresponding proteins (turnover differences) were calculated using N/P ratios or turnover rates. **b** Same as in panel a, but for proteins of the 19S regulatory subunit. **c** Positioning of some of the PTMs that significantly associated with protein turnover (PDB 5LE5[1]). Only the α-ring of the 20S core particle is displayed viewing from the direction of the 19S regulatory particle as shown by the small representation of half of a 30S proteasome. Source data are provided as a Source Data file.

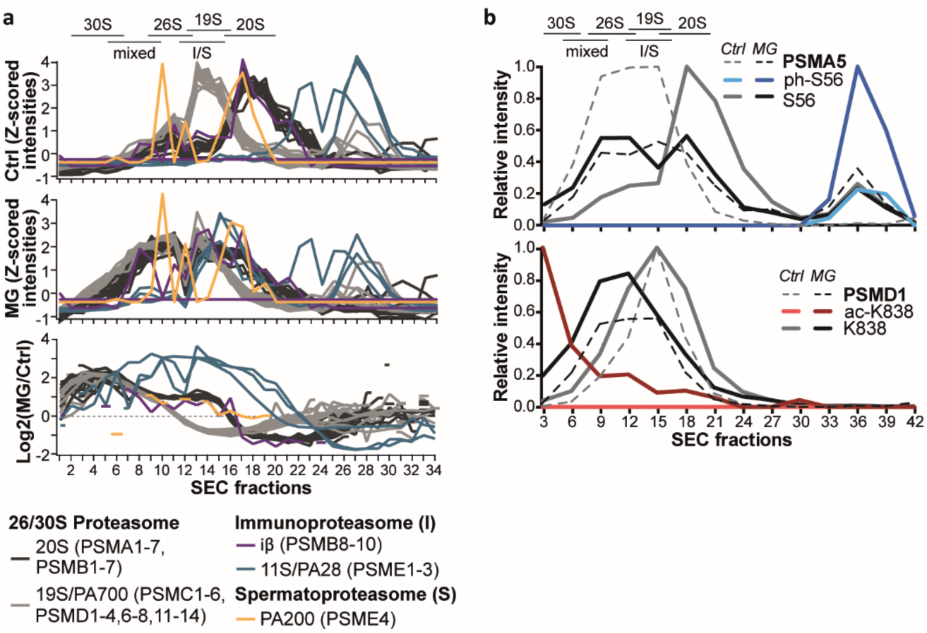

Supplementary Fig. 7 | PTMs in different complex and monomer fractions of the proteasome. a Size exclusion chromatography (SEC) profiles of proteasomal subunits in HeLa cells upon proteasome inhibition. The upper and middle panel show profiles from control and MG132-treated cells (MG, 1 µM, 16h), respectively. The lower panel displays changes upon proteasome inhibition as log2 ratios. Fractions containing proteasome complexes are indicated based on extrapolation of lower molecular weight markers (mixed: 20S+19S+11S or 20S+19S+PA200). **b** SEC profiles for proteins, modified and unmodified sites of PSMA5 and PSMD1 with and without proteasome inhibition. Peptide profiles were acquired via targeted parallel-reaction monitoring assays using pooled SEC fractions, while protein intensities from whole proteome measurements were combined *in silico* for every three adjacent fractions. Source data are provided as a Source Data file.

a

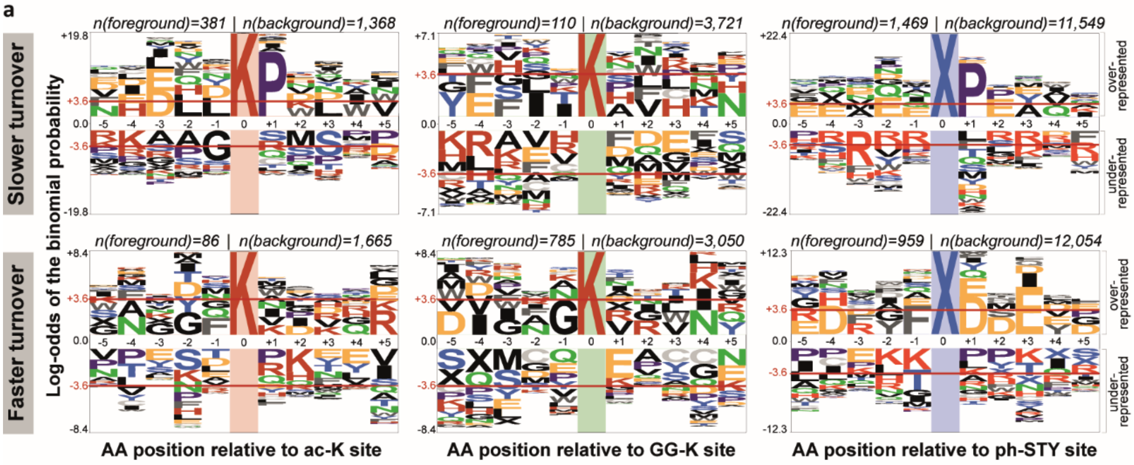

b

Slower pST motif	Score	Fold increase	Foreground matches	Foreground size	Background matches	Background size
...E.[ST]P....	22.1	2.3	74	1457	250	11497
.....[ST]PE...	15.9	1.8	95	1383	431	11247
.....[ST]PP...	13.3	1.7	124	1288	613	10816
.....[ST]P....	5.5	1.2	553	1164	4177	10203
**Slower acK motif**						
.....KP....	15.4	3.9	50	381	46	1370
..E..K.....	6.1	2.2	45	331	82	1324
..D..K.....	5.2	3.2	20	286	27	1242
**Faster pST motif**						
.....[ST]..E..	6.9	1.5	159	956	1319	11993

c

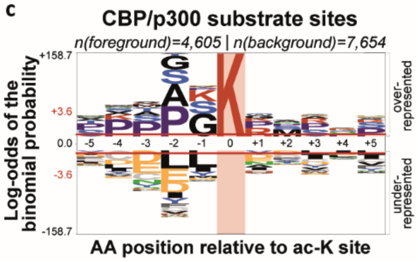

Supplementary Fig. 8 | Sequence motif analyses of modification sites with differential turnover. a Probability logos of ac-K (left), GG-K (middle) and ph-ST sites (right) that showed slower (top) or faster (bottom) turnover (red horizontal line: Bonferroni corrected p-value of 0.05 indicating significant over- or underrepresentation). Logos were generated using pLogo[2] (https://plogo.uconn.edu/). The background was defined by all sites quantified in this study. **b** Motifs extracted from modified peptides with measured slower or faster turnover using the motifX algorithm via the R package rmotifx. **c** Probability logo of substrates of the lysine acetyltransferase (KAT) CBP/p300. Substrate sites were defined as sites that showed a two-fold down-regulation after CBP/p300 inhibition or two-fold up-regulation after CBP/p300 transfection as reported by Weinert *et al.* (red horizontal line: p-value of 0.05).

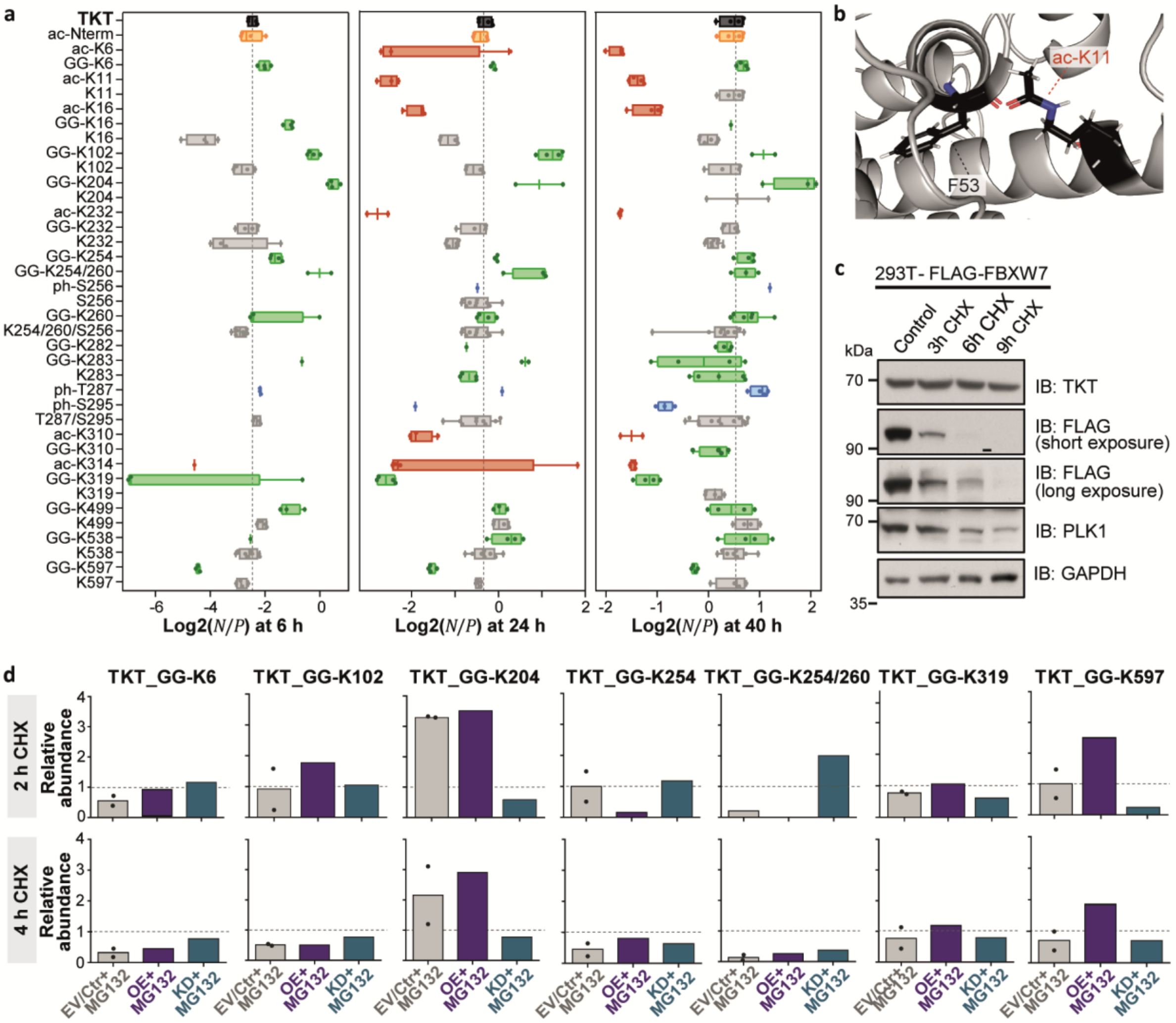

Supplementary Fig. 9 | Measured TKT turnover. a Tukey-boxplots of log2 N/P ratios of TKT and all quantified PTMs (box borders: 1st and 3rd quartile; lines in boxes: medians; whiskers: ranging to greatest value within 1.5× interquartile range; dots: replicates; dashed line: median of protein N/P). **b** Clash of the acetyl group on K11 with the backbone carbonyl group of F53 (PDB 3MOS[2]). The PyMol plugin PyTMs was used to add the acetyl moiety to the lysine residue. **c** Western blot of CHX chases in cells transfected with FLAG-FBXW7. Results shown are representative of 2 replicates. **d** Bar charts illustrating the response of ubiquitination for individual lysine sites of TKT after 2 h (top) and 4 h (bottom) CHX treatment and proteasome inhibition using MG132 (EV: empty vector; CTR: control siRNA). Di-gly peptide profiles were acquired via targeted parallel-reaction monitoring assays and relative intensities were calculated using appropriate controls (EV/CTR for EV/CTR+MG132, OE for OE+MG132, CTR+MG132 for KD+MG132). Points represent the EV and CTR sample (n=2). Source data are provided as a Source Data file.

Supplementary Discussion

Two isotopic labelling workflows were employed for the study of PTM-resolved turnover. Using the dSILAC-TMT method, we measured label incorporation and loss in a time-dependent manner and estimated turnover rates of peptidoforms via curve fitting. In contrast, N/P ratios obtained in single pulse experiments provided the relative pace of turnover within a certain pulse time-point. Hence, no curve fitting and filtering were necessary, which simplified overall data processing. However, the observable turnover range within an individual time-point was more limited since relatively higher or lower turned over peptidoforms more likely escaped analysis. This was mostly a result of missing quantitative data for one of the two SILAC channels. For dSILAC-TMT time-course experiments, extreme turnover behaviour only affected the likelihood of fragmenting (by mass spectrometry) and obtaining a curve for exclusively the newly synthesized (very high turnover) or the pre-existing peptide species (very low turnover) but did not impair the general probability of detection.

While those peptidoforms with considerably faster or slower turnover may be the most interesting ones, missing values cannot be rescued reliably in data of individual pulse time-points because data imputation may strongly influence and bias quantification[3]. The constraint of such missing quantitative data was particularly apparent for the 1 h and 40 h time-points, which showed overall decreased numbers of peptides quantified in both SILAC labelling states. Notably, this also entailed the quantification of more distinct selections of peptidoform across the individual time-points than within replicates of the same pulse length. This is important to mention since the subset of peptides identified for a protein or protein group will affect the overall assessment of protein turnover. Similarly, the identification of peptidoforms with vastly differing turnover can be impeded within the same pulse time-point. The greater their difference, the less likely they are quantified in both labelling states. This may also explain the comparably small number of ac-K and GG-K sites available for the investigation of their interplay within the same pulse length. In earlier time-points, more ubiquitin-remnant peptides

were quantified, while later time-points showed increasing quantifications of ac-K peptidoforms.

We excluded the 1 h pulse data from further analysis due to the poor correlations across label swap experiments. These were caused by distinct subpopulations of peptides that deteriorated the otherwise excellent concordance of remaining peptides. While this was most notable for 1 h pulse data, the other pulse time-points showed similar, albeit less profound biases. Others have already reported quantitative discordance between label-swap experiments in cross-linking experiments as a result of large peptide mass, low intensity, and heavily overlapping isotope clusters of light and heavy signals[4]. Besides, quantification accuracy may be impaired by isotope impurities of SILAC amino acids, but this is unlikely to produce such strong effects. Further systematic analyses are required to clarify the chief cause for erroneous quantification in dSILAC experiments with very short or long pulses.

To improve the quality of dSILAC-TMT data and the fraction of successful curve fits, we adjusted two major points in our data processing pipeline compared to the initial establishment of the dynamic/pulsed SILAC-TMT approach[5]. First, co-isolated intensities from peptides with opposing label characteristics (loss vs. incorporation) were removed *in silico*. Several approaches have been described already for computational elimination of co-isolated intensities, but they rely on either spike-in standards[6] or the estimation of the precursor intensity fraction within the isolation window[7]. Further, they assume a constant, co-isolated background across conditions. The later presumption renders such approaches inapplicable to dSILAC-TMT data since co-isolated peptides in such experiments always exhibit decreasing or increasing intensities across time-points. Hence, instead of removing a constant background, we subtracted average label incorporation or loss curves from curves that showed residual intensities in the infinite h or 0 h channels, respectively. Of note, co-isolation of peptides with the same labelling behaviour (e.g. both indicating label loss) would not result in a measurable ratio compression and thus cannot be corrected. In such cases, however, quantification accuracy would only be influenced to a minor degree. As expected, the removal

of co-isolated intensities increased the number of successful curve fits especially for low abundant, ac-K and ph-Y peptidoforms. As a second step, we corrected peptide intensities for abundance changes during the time-course of the experiment. Thereby, we improved the accordance with steady-state assumptions that underlie the curve-fitting algorithm (see methods and Zecha *et al.*[5]). Importantly, such corrections must be assessed carefully to minimize potential processing artefacts. For example, if only one entry for each curve is available, label incorporation and loss curves would be inevitably forced to yield identical turnover rates after correction. This would artificially improve correlations when comparing label loss and incorporation. Therefore, we decided to require at least 4 curves per peptidoform and calculate correction factors globally and not separately for each replicate. Thus, we corrected primarily abundance changes that were reproducible across replicates. Consequently, only a fraction of peptides was subjected to such intensity adjustments, but the overall correlation across replicates still increased considerably.

In contrast to dSILAC experiments, the time-course characteristic of dSILAC-TMT data required more sophisticated data processing. Noteworthy, however, curve fitting and filtering constitute an additional level of quality control. We further decreased false identifications and poor quantifications using the prior knowledge about which peptide (depending on the SILAC label) should exhibit label incorporation and which one should resemble label loss. Peptidoforms that did not show the expected quantitative trend were automatically filtered out by our KRAB criteria. However, peptides that did not adhere well enough to the assumptions underlying the curve-fitting algorithm despite representing valid identifications were equally removed. Hence, we limited the observable turnover behaviour to *a priori* suppositions that may not apply to all observable turnover behaviours. The high proportion of evidence entries that did not pass the KRAB filter criteria especially for acK peptidoforms suggested that this might have been a prevalent issue. Hence, alternative fitting algorithms may recover even more of the time-course data and enable an extended analysis in the future.

Supplementary References

1. Mitschke, L. et al. The crystal structure of human transketolase and new insights into its mode of action. *J Biol Chem* **285**, 31559-31570 (2010).
2. O'Shea, J.P. et al. pLogo: a probabilistic approach to visualizing sequence motifs. *Nature Methods* **10**, 1211-1212 (2013).
3. O’Connell, J.D., Paulo, J.A., O’Brien, J.J. & Gygi, S.P. Proteome-Wide Evaluation of Two Common Protein Quantification Methods. *Journal of Proteome Research* **17**, 1934-1942 (2018).
4. Chen, Z.A., Fischer, L., Cox, J. & Rappsilber, J. Quantitative Cross-linking/Mass Spectrometry Using Isotope-labeled Cross-linkers and MaxQuant. *Molecular & Cellular Proteomics* **15**, 2769-2778 (2016).
5. Zecha, J. et al. Peptide Level Turnover Measurements Enable the Study of Proteoform Dynamics. *Molecular & Cellular Proteomics* **17**, 974-992 (2018).
6. Karp, N.A. et al. Addressing Accuracy and Precision Issues in iTRAQ Quantitation. *Molecular & Cellular Proteomics* **9**, 1885-1897 (2010).
7. Savitski, M.M. et al. Measuring and Managing Ratio Compression for Accurate iTRAQ/TMT Quantification. *Journal of Proteome Research* **12**, 3586-3598 (2013).
