# Supplementary material for: Linking post-translational modifications and protein turnover by site-resolved protein turnover profiling
Source: Nat Commun. 2022 Jan 10;13:165. doi: 10.1038/s41467-021-27639-0 (PMC8748498; doi:10.1038/s41467-021-27639-0)
Supplement: Supplementary file 3 — Description of Additional Supplementary Files [file 41467_2021_27639_MOESM3_ESM.docx]

**Description of Additional Supplementary Files**

**File Name:** Supplementary Data 1

**Description:** Processed turnover data for peptidoforms and proteins. Contains processed data from MaxQuant searches of individual time-points using the dSILAC workflow and of the pulse time-series using the dSILAC-TMT method.

**File Name:** Supplementary Data 2

**Description:** Results of Student's t-tests. Provides results of two-sided Student's t-tests that were performed to find statistically significant differences between the turnover of peptidoforms and their corresponding protein (pep-prot) or their unmodified counterpart peptides (mod-counter).

**File Name:** Supplementary Data 3

**Description:** Combined information about peptidoforms and modification sites from all datasets. Contains combined information about peptidoforms and modified sites from all 4 datasets (dSILAC 6 h, 24 h, 40 h, dSILAC-TMT time-series) and replicates. Including average fold changes between peptidoforms or sites to their corresponding proteins or counterpart peptides/sites, their statistical significance, and functional annotations.

**File Name:** Supplementary Data 4

**Description:** Fisher's Exact tests. Provides results of Fisher's exact tests for the enrichment analyses of functional categories within peptidoforms and sites with significant turnover differences, and within lysine sites that were identified in both an acetylated and a ubiquitinated state.

**File Name:** Supplementary Data 5

**Description:** Size exclusion experiments. Contains results of MS measurements of fractions from the size exclusion chromatography (SEC) of PRMI8226 and HeLa cells after proteasome inhibition with bortezomib (BTZ) or MG132, respectively.

**File Name:** Supplementary Data 6

**Description:** Ac-GG-K pairs. Provides turnover information on all lysine residues for which turnover was determined in an acetylated, di-Gly (ubiquitin-remnant), and unmodified state including functional annotations.

**File Name:** Supplementary Data 7

**Description:** CHX-chases. Contains the results of parallelreaction-monitoring assays for potential FBXW7 phosphodegrons & diGly sites on TKT in cycloheximid treated HEK293T cells.

**File Name:** Supplementary Data 8

**Description:** Complete list of oligonucleotides used. Lists nucleotide sequences of FBXW7 overexpression constructs and siRNAs.
