# Supplementary material for: Linking post-translational modifications and protein turnover by site-resolved protein turnover profiling
Source: Nat Commun. 2022 Jan 10;13:165. doi: 10.1038/s41467-021-27639-0 (PMC8748498; doi:10.1038/s41467-021-27639-0)
Supplement: Supplementary file 12 — Reporting Summary [file 41467_2021_27639_MOESM12_ESM.pdf]

nature research

Corresponding author(s): Bernhard Kuster

Last updated by author(s): Nov 14, 2021

Reporting Summary

Nature Research wishes to improve the reproducibility of the work that we publish. This form provides structure for consistency and transparency in reporting. For further information on Nature Research policies, see our Editorial Policies and the Editorial Policy Checklist.

Statistics

For all statistical analyses, confirm that the following items are present in the figure legend, table legend, main text, or Methods section.

n/a	Confirmed	
☐	☒	The exact sample size (*n*) for each experimental group/condition, given as a discrete number and unit of measurement
☐	☒	A statement on whether measurements were taken from distinct samples or whether the same sample was measured repeatedly
☐	☒	The statistical test(s) used AND whether they are one- or two-sided<br>*Only common tests should be described solely by name; describe more complex techniques in the Methods section.*
☒	☐	A description of all covariates tested
☐	☒	A description of any assumptions or corrections, such as tests of normality and adjustment for multiple comparisons
☐	☒	A full description of the statistical parameters including central tendency (e.g. means) or other basic estimates (e.g. regression coefficient) AND variation (e.g. standard deviation) or associated estimates of uncertainty (e.g. confidence intervals)
☐	☒	For null hypothesis testing, the test statistic (e.g. *F*, *t*, *r*) with confidence intervals, effect sizes, degrees of freedom and *P* value noted<br>*Give P values as exact values whenever suitable.*
☒	☐	For Bayesian analysis, information on the choice of priors and Markov chain Monte Carlo settings
☒	☐	For hierarchical and complex designs, identification of the appropriate level for tests and full reporting of outcomes
☒	☐	Estimates of effect sizes (e.g. Cohen's *d*, Pearson's *r*), indicating how they were calculated

Our web collection on statistics for biologists contains articles on many of the points above.

Software and code

Policy information about availability of computer code

Data collection	Mass spectrometry data was collected using commercial software associated with the mass spectrometers (XCalibur, Thermo Scientific, respective most recent versions at the time of data acquisition).
Data analysis	Peptide identification and quantification was performed using MaxQuant v1.6.0.1. or Skyline v20.2.0.286. Spectra prediction was performed with the Prosit 2019 algorithm. R v3.4.1 or v3.6.0, RStudio v1.3.1093, Perseus v1.6.2.3, Microsoft Excel 2013, GraphPad Prism 5, and Venn Diagram Plotter v1.5 were used for further data processing and/or plotting. Protein crystal structures were visualized in PyMOL v2.4.1. Motif enrichment analyses were performed using the R package rmotifx and visualization was performed using pLogo accessible via https://plogo.uconn.edu/.

For manuscripts utilizing custom algorithms or software that are central to the research but not yet described in published literature, software must be made available to editors and reviewers. We strongly encourage code deposition in a community repository (e.g. GitHub). See the Nature Research guidelines for submitting code & software for further information.

Data

Policy information about availability of data

All manuscripts must include a data availability statement. This statement should provide the following information, where applicable:

- Accession codes, unique identifiers, or web links for publicly available datasets
- A list of figures that have associated raw data
- A description of any restrictions on data availability

The MS proteomics raw data and MaxQuant search results including the fasta file from the Swiss-Prot database have been deposited to the ProteomeXchange Consortium via the PRIDE partner repository under the accession codes PXD023218 [http://proteomecentral.proteomexchange.org/cgi/GetDataset?ID=PXD023218] (dSILAC, dSILAC-TMT, and SEC experiments) and PXD023234 [http://proteomecentral.proteomexchange.org/cgi/GetDataset?ID=PXD023234] (SEC and CHX PRM

assays). Raw data and results of the PRM assays can also be examined on the Panorama Public website [https://panoramaweb.org/SPOT.url] (SEC and CHX experiments). Normalized and further processed data such as turnover rates and N/P ratios and nucleotide sequences are provided in the Supplementary Data. In addition, all analyzed data of modified and counterpart sites can be viewed and explored via the SPOT web tool [spot.proteomics.wzw.tum.de]. Source data of all figures are provided with this paper in the Source Data file. Previously published potein crystal structures can be obtained from the RCSB protein databank website (PDB IDs: 5LE5, 3B97, 3MOS).

Field-specific reporting

Please select the one below that is the best fit for your research. If you are not sure, read the appropriate sections before making your selection.

☒ Life sciences ☐ Behavioural & social sciences ☐ Ecological, evolutionary & environmental sciences

For a reference copy of the document with all sections, see nature.com/documents/nr-reporting-summary-flat.pdf

Life sciences study design

All studies must disclose on these points even when the disclosure is negative.

Sample size

In turnover analyses, no groups of conditions are compared to each other. Therefore, no sample-size calculation was performed. We performed 4 independent cell culture replicates for all pulse experiments to enable statistical analysis of turnover differences between individual peptides and their corresponding protein. Size exclusion chromatography (SEC) was performed for two different cell lines (biological replicates) to assess biological reproducibility. FBXW7 knockdown and overexpression results are based on duplicate experiments including respective controls.

Data exclusions

The 1 h SILAC pulse was excluded from analyses because it exhibited substantial amino acid recycling and inferior correlations between SILAC label swap experiments (replicates) implicating erroneous turnover estimations. This is probably a result of a bias in the data analysis software for extreme SILAC ratios. One FBXW7 knockdown replicate aligned exactly with the results of the two other replicates for the phosphopeptide of TKT, but the positive control (phosphopeptide of UNG) was not showing the behaviour expected based on literature reports. Thus, this replicate was excluded from the analysis. One FBXW7 overexpression replicate suffered from irreproducible phosphopeptide enrichment due to inconsistent washing in the automated workflow and was therefore also excluded from the analysis.

Replication

We conducted 4 independent cell culture replicates for all pulse experiments to enable statistical analysis and evaluate reproducibility of findings. The assessment of repeatability of turnover measurements is an integral part of the manuscript and showed good correlation and reproducibility between experiments. The authors note, however, that replicates of OMICS experiments and cell culture experiments never lead to exactly the same results (single proteins and peptides in the current case). The SEC experiments using two cell lines (biological duplicates) also produced comparable results indicating biological reproducibility. For FBXW7 overexpression and knockdown experiments, 3 independent replicates were analysed to assess reproducibility. One of each showed differing results to some extent due to impaired phosphoenrichment efficiency and a negative 'positive control' (see also data exclusion).

Randomization

The experiments/measurements did not require randomization and control of covariates since they did not include any human subjects and for the following additional reasons: Turnover experiments do not include different treatments or cell lines that could be randomized. Time-points of dSILAC-TMT experiments were multiplexed using tandem-mass-tags enabling measurement of all time-points in parallel precluding randomization. Time-points of dSILAC experiments were analysed independently. Thus they did not require randomization. For SEC experiments, treatment conditions were quantified using the isotopic labeling technique SILAC which allows for sample mixing and simultaneous data acquisition precluding randomization. Data from the two cell lines of SEC experiments were analysed independently. Thus they did not require randomization.

Blinding

In this work, either no groups of conditions are compared to each other or data acquisition of treated and non-treated samples took place simultaneously. Therefore, the study did not require blinding.

Reporting for specific materials, systems and methods

We require information from authors about some types of materials, experimental systems and methods used in many studies. Here, indicate whether each material, system or method listed is relevant to your study. If you are not sure if a list item applies to your research, read the appropriate section before selecting a response.

Materials & experimental systems

n/a	Involved in the study	
☐	☒	Antibodies
☐	☒	Eukaryotic cell lines
☒	☐	Palaeontology and archaeology
☒	☐	Animals and other organisms
☒	☐	Human research participants
☒	☐	Clinical data
☒	☐	Dual use research of concern

Methods

n/a	Involved in the study	
☒	☐	ChIP-seq
☒	☐	Flow cytometry
☒	☐	MRI-based neuroimaging

Antibodies

Antibodies used	anti-FLAG: Monoclonal ANTI-FLAG® M2 antibody produced in mouse, Sigma, #F3165, clone M2, lot# SLBS3530V<br>anti-TKT: Anti-Transketolase Antibody (H-7), Santa Cruz Biotechnology, #sc-390179, clone H-7, lot# A2617<br>anti-GAPDH: Anti-GAPDH Antibody (0411), Santa Cruz Biotechnology, #sc-47724, clone 0411, lot# J1119<br>anti-PLK1: PLK1 Antibody Cocktail, Thermo Fisher Scientific; #33-1700, lot# VH313459
Validation	Based on the manufacturer websites, all used antibodies are recommended for human samples on western blots. According to the manufacturer, the anti-Flag antibody detects a single band of protein on a western blot from an E. coli crude cell lysate and has been cited in >6,100 peer-reviewed publications including for western blotting of human samples (papers can be accessed at https://www.sigmaaldrich.com/DE/en/search/f3165?focus=papers&page=1&perPage=30&sort=relevance&term=F3165&type=citation_search). The anti-TKT antibody detects a single band on western blots using rat and mouse tissue extracts (manufacturer statement), has been cited in at least 8 publications, and has a 4.9 star customer rating (see https://www.scbt.com/de/p/transketolase-antibody-h-7 for manufacturer statement and links to papers). The anti-GAPDH antibody detects a single band on western blots using human cell lines (manufacturer statement), has been cited >2300 times, and has an average 4.7 star rating from users (see https://www.scbt.com/de/p/gapdh-antibody-0411 for manufacturer statement and links to papers). The anti-PLK1 antibody cocktail has been used in at least 45 publications (https://www.thermofisher.com/antibody/product/PLK1-Antibody-clone-PL6-PL2-Cocktail/33-1700). Based on the figures provided by the manufacturer, the PLK antibody cocktail produces (at least) 2 bands which was confirmed by our western blot experiments. All other antibodies produced single bands in our western blot experiments.

Eukaryotic cell lines

Policy information about cell lines

Cell line source(s)	HeLa, RPMI8226, and HEK293T were obtained from ATCC (product numbers #CCL-2, #CCL-155, and #CRL-3216)
Authentication	HeLa and RPMI8226 cell lines were authenticated with 98 and 96 % identity, respectively, using Multiplex Cell Authentication (MCA; based on Single Nucleotide Polymorphism (SNP)-Profiling) performed by Multiplexion (HeLa SNP: AAATAAAATTAAATATATATTTATTTAAATWTATATATTTTTAAAATT; RPMI8226 SNP: AATTTTAAAAATTTAATTAAATATTTAAAAAAATAAAATTTTAATTAT). HEK293T cells have not recently been authenticated.
Mycoplasma contamination	Cell lines were negative for mycoplasma contamination in this project.
Commonly misidentified lines (See ICLAC register)	No commonly misidentified cell lines were used.
